# MicroRNAs as Therapeutic Targets and Clinical Biomarkers in Atherosclerosis

**DOI:** 10.3390/jcm8122199

**Published:** 2019-12-13

**Authors:** Emma L. Solly, Catherine G. Dimasi, Christina A. Bursill, Peter J. Psaltis, Joanne T. M. Tan

**Affiliations:** 1 Vascular Research Centre, Heart and Vascular Health Program, Lifelong Health Theme, South Australian Health and Medical Research Institute, Adelaide SA 5000, Australia; emma.solly@sahmri.com (E.L.S.); catherine.dimasi@sahmri.com (C.G.D.); christina.bursill@sahmri.com (C.A.B.); peter.psaltis@sahmri.com (P.J.P.); 2 Adelaide Medical School, University of Adelaide, Adelaide SA 5005, Australia

**Keywords:** inflammation, oxidative stress, angiogenesis, endothelial dysfunction, smooth muscle cells, foam cell formation, plaque stability, plaque rupture, vasa vasorum

## Abstract

Atherosclerotic cardiovascular disease remains the leading cause of morbidity and mortality worldwide. Atherosclerosis develops over several decades and is mediated by a complex interplay of cellular mechanisms that drive a chronic inflammatory milieu and cell-to-cell interactions between endothelial cells, smooth muscle cells and macrophages that promote plaque development and progression. While there has been significant therapeutic advancement, there remains a gap where novel therapeutic approaches can complement current therapies to provide a holistic approach for treating atherosclerosis to orchestrate the regulation of complex signalling networks across multiple cell types and different stages of disease progression. MicroRNAs (miRNAs) are emerging as important post-transcriptional regulators of a suite of molecular signalling pathways and pathophysiological cellular effects. Furthermore, circulating miRNAs have emerged as a new class of disease biomarkers to better inform clinical diagnosis and provide new avenues for personalised therapies. This review focusses on recent insights into the potential role of miRNAs both as therapeutic targets in the regulation of the most influential processes that govern atherosclerosis and as clinical biomarkers that may be reflective of disease severity, highlighting the potential theranostic (therapeutic and diagnostic) properties of miRNAs in the management of cardiovascular disease.

## 1. Introduction

Coronary artery disease (CAD) involves the narrowing or hardening of arteries, restricting blood flow to the heart and resulting in cardiovascular diseases such as angina, myocardial infarction or even sudden death. CAD is driven by atherosclerosis, a multi-faceted disease that develops over several decades that is mediated by a complex interplay between a plethora of cellular mechanisms and interactions between endothelial cells (ECs), smooth muscle cells (SMCs) and inflammatory cells. These mechanisms drive a chronic inflammatory milieu that promotes plaque development and progression, in which lipid build-up in the arteries leads to plaque formation. Despite significant research and therapeutic advances, the impact of atherosclerosis on cardiovascular disease is not fully resolved, highlighting the need for alternate therapies and improved ways to better detect disease progression. MicroRNAs (miRNAs), small non-coding RNAs that simultaneously control the expression of multiple genes, are emerging as powerful clinical biomarkers and therapeutic targets for multi-faceted diseases [1]. miRNA targeting drugs are showing promise in Phase I and II clinical trials in a wide range of diseases including scleroderma, cancer and hepatitis C virus [2], making them the leading next generation biopharmaceuticals [1]. Furthermore, circulating miRNAs have emerged as a new class of disease biomarkers [3]. This review focusses on recent insights into the potential role of miRNAs both as therapeutic targets in the regulation of the most influential processes that govern atherosclerosis and as clinical biomarkers that may be reflective of disease severity, highlighting the potential therapeutic and diagnostic properties of miRNAs in the future management of cardiovascular disease.

## 2. MicroRNA Biology

During canonical biogenesis, miRNAs are transcribed by RNA polymerase II, producing a double-stranded hairpin primary (pri)-miRNA transcript [4]. pri-miRNAs are then cleaved by the enzyme Drosha generating a short hairpin structure termed pre-miRNA. Pre-miRNA is exported to the cytoplasm, where the RNase III endonuclease Dicer removes the terminal loop of pre-miRNA producing mature miRNA duplex strands [4]. Each strand is termed either −5p or −3p, representative of the 5’ or 3’ directionality of the strand. Either strand can be loaded onto the protein Argonaute to form the RNA-induced silencing complex (RISC). The proportion of −5p to −3p in RISC is dependent on the thermodynamic stability of the strands and the less stable of the two strands will be degraded [5]. However, the proportion of −5p to −3p can be either equal or, depending on the cell type, may favour one strand over the other. Additionally, the functionality of each strand can differ, and RISC may be directed towards divergent gene targets depending on the proportion/presence of the −5p or −3p strands [6,7]. miRNAs then mediate gene expression through translational repression or targeted degradation by binding through complementary base pairing within the 3’-UTR of their mRNA targets. miRNAs have been shown to play a role in regulating numerous cellular processes and have been shown to contribute to disease progression, regulating up to 60% of all genes in the human genome [8]. Furthermore, miRNAs and pre-miRNAs can be released from the cell into the bloodstream and are extremely stable in the extracellular environment, either in free form or trapped in circulating microvesicles, exosomes, high-density lipoproteins (HDL) or protein complexes, where they can be taken up within tissues by cell-to-cell communication [8]. They continue to inspire a myriad of research and are emerging as therapeutic targets and clinical biomarkers for personalised medicine in complex diseases.

## 3. MiRNAs in Cholesterol Homeostasis and Reverse Cholesterol Transport

**Figure 1:**
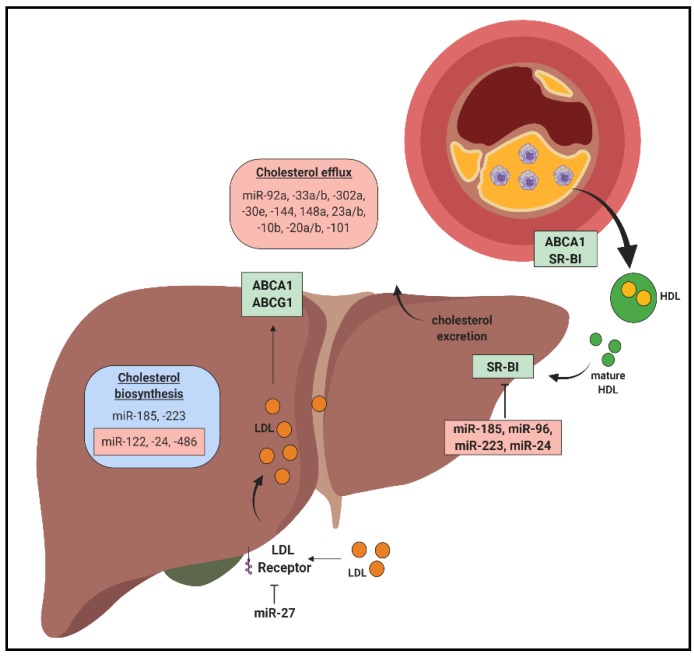
MiRNAs in cholesterol homeostasis and reverse cholesterol transport. In the liver, miRNAs target critical mediators involved in cholesterol biosynthesis, exerting positive/atheroprotective (blue) or negative/atherogenic (red) effects. miRNAs target cholesterol uptake into the liver by inhibiting expression of cholesterol transporters scavenger receptor BI (SR-BI) and low density lipoprotein receptor (LDLR). MiRNAs also have a significant impact in mediating cholesterol efflux capacity from the atherosclerotic plaque.

Cholesterol is an important player in every stage of atherosclerosis development [9]. In the circulation, cholesterol is carried on lipoproteins, in which low-density lipoproteins (LDL) deliver and high-density lipoproteins (HDL) remove cholesterol from cells and tissues to mediate cholesterol homeostasis. Excess circulating LDL levels contribute to lipid build-up in regions susceptible to plaque formation. LDL is prone to oxidative and enzymatic modification, predisposing it to unregulated uptake into macrophages, leading to the formation of atherosclerotic lesions [10]. Conversely, reduced cholesterol efflux capacity is a robust predictor for atherosclerosis in humans. miRNAs are known to be involved in maintaining the balance between atherogenic LDL and atheroprotective HDL levels and function (Figure 1).

The liver plays a critical role in the production and clearance of lipoproteins. Numerous hepatic-enriched miRNAs are known to regulate lipoprotein metabolism. miR-122 is a crucial regulator of cholesterol and fatty acid synthesis and has been highlighted as a promising target for lowering plasma cholesterol in humans [11]. Cholesterol synthesis is tightly regulated by sterol regulatory element binding proteins (SREBP) which detect low cellular cholesterol levels, promoting its synthesis and uptake. miR-185 reduces de novo cholesterol synthesis by downregulating SREBP2 and LDL receptor (LDLR) [12]. Furthermore, SREBP1 increases miR-185 levels [12], suggesting that miR-185 expression is tightly regulated to control intracellular cholesterol homeostasis. Paradoxically, miR-185 also represses the expression of KH-type splicing regulatory protein (KSRP), a negative regulator of LDLR, providing novel insights into the miR-185-mediated regulatory network responsible for the regulation of hepatic LDLR expression [13]. Importantly, inhibition of miR-185 in vivo reduced plasma cholesterol levels and decreased plaque area in mice [13]. miR-33a/b is co-expressed with SREBP1/2 and miR-33a dysregulation is thought to contribute to atherosclerosis by promoting lipid build-up and cholesterol retention in macrophages through the ATP binding cassette (ABC) transporter ABCA1 [14]. The close regulation of miR-33a/b and miR-185 with SREBP1/2 suggest that miR-33a/b and miR-185 play a crucial role in cholesterol homeostasis and that their dysregulation may be an early event in atherosclerotic progression.

miR-223 inhibits genes involved in cholesterol biosynthesis (e.g., 3-Hydroxy-3-methylglutaryl-CoA synthase 1 (HMGCS1) and methylsterol monooxygenase 1 (SC4MOL)) and HDL uptake (scavenger receptor-BI, SR-BI), and miR-223^−/−^ mice have elevated hepatic and plasma total and HDL cholesterol levels [15]. miR-24 has also been shown to aggravate atherosclerosis by inhibiting genes involved in lipogenesis (e.g., insulin-induced gene 1 (INSIG1), an inhibitor of lipogenesis) [16] and HDL uptake (SR-BI) [17]. In vivo, miR-24 administration decreased hepatic SR-BI expression and promoted atheromatous plaque formation in atherosclerotic-prone apolipoprotein E-null (*Apoe*^−/−^) mice [17]. Inhibition of miR-486 and miR-92a decreased liver and plasma cholesterol by targeting SREBP1 and ABCG4, respectively [18]. miR-27 also decreased intracellular cholesterol uptake and increased plasma cholesterol by inhibiting LDLR expression [19,20] and molecules involved in efficient LDL endocytosis (e.g., low density lipoprotein receptor adaptor protein 1, LDLRAP1; LDL receptor related protein 6, LRP6) [20]. miR-30c reduces hyperlipidaemia by inhibiting microsomal triglyceride transfer protein (MTTP) to restrict assembly and secretion of apoB lipoproteins and lysophosphatidylglycerol acyltransferase 1 (LPGAT1) to inhibit de novo lipogenesis, leading to decreased levels of plasma total and LDL cholesterol [21]. miR-30c overexpression in *Apoe*^−/−^ mice mitigated atherosclerosis without inducing steatosis, an undesirable side effect associated with conventional MTTP inhibitors [21].

miRNAs that regulate reverse cholesterol transport have also been explored for their therapeutic potential in promoting the regression of atherosclerotic plaques. miR-33a and miR-33b inhibit the expression of the cholesterol transporters ABCA1 and ABCG1 in macrophages resulting in reduced cholesterol efflux to HDL [14,22,23]. Inhibition of miR-33a/b enhanced cholesterol efflux and regression of atherosclerotic plaques in *Ldlr*^−/−^ mice [24] and improved lipid profiles in non-human primates [25,26]. Other miRNAs including miR-144, miR-27a/b, miR-302a, miR-148a, miR-92a, miR-30e, miR-101, miR-23a-5p, miR-20a/b and miR-10b also target cholesterol transporters (LDLR and ABCA1) [19,27,28,29,30,31,32,33,34], highlighting their potential in regulating atherosclerosis. Cholesterol-loaded mature HDL facilitates the removal or redistribution of cholesterol via its uptake in the liver through SR-BI. In atherosclerosis, miR-185, miR-96, miR-223 and miR-24 target hepatic SR-BI to suppress HDL uptake in the liver, preventing cholesterol excretion by the liver [15,17,30,35]. miR-24 was further shown to contribute to plaque progression, promoting lipid accumulation in the liver [17,36]. Overall, these studies demonstrate that multiple miRNAs can contribute to the burden of lipid build-up in the arteries via different mechanisms, and those with multi-faceted effects on these processes represent the most fundamental regulators that likely contribute to disease progression.

Evidence suggests that postprandial chylomicron (CM) and triglyceride-rich very low-density lipoprotein (VLDL) particles also play an important part in atherosclerotic plaque development [37]. While miRNAs that mediate the ability of HDL to uptake and transport triglycerides has been extensively investigated, it is currently unknown to what extent miRNAs are able to regulate VLDL in the postprandial state. This is of importance given that VLDL particles comprise 80% of triglyceride-rich lipoprotein (TGRL) remnants [38]. Studies have reported that miR-30c mediates apoB lipoprotein assembly [21], and inhibition of miR-33 lowers VLDL-triglyceride levels in non-human primates [25]. However, whether these miRNAs contribute to dysregulated postprandial lipemia in atherosclerosis is currently unknown. Clinical data show a correlation between postprandial lipoproteins and the presence/progression of CAD [39]. Furthermore, studies show that non-fasting and postprandial lipid parameters may better predict CVD risk compared to traditional fasting measurements [40,41]. Mechanistic studies demonstrate that, although CM and VLDL are too large for passage into the arterial intima, their smaller cholesterol-enriched remnants can penetrate the vascular subendothelial space, driving a pro-inflammatory state in the endothelium and promoting monocyte recruitment and monocyte/macrophage accumulation in atherosclerotic plaques [39,42]. Ex vivo analysis of isolated TGRL in subjects fed a high-fat diet showed that TGRL bias the endothelial pro-inflammatory response via post-transcriptional editing of vascular cell adhesion molecule (VCAM)-1 through the action of miR-126 [43]. Interestingly, subjects with an anti-inflammatory response to a meal produced TGRL that increased endothelial miR-126 activity concomitant with reduced VCAM-1 levels and diminished monocyte arrest [43], highlighting a direct role for miR-126 in response to postprandial TGRL. Recently, miR-206-3p, miR-409-3p and miR-27b-5p were found to be increased in human exosomes in response to fatty meals [44]. Pathway analysis suggests that these miRNAs target genes are involved in different processes including insulin signalling, lipid metabolism, angiogenesis and inflammation, highlighting the potential role of postprandial circulating miRNAs as mediators of the molecular response to postprandial lipemia and their influence on atherosclerosis.

## 4. MiRNAs in Atherosclerotic Plaque Initiation and Progression

**Figure 2:**
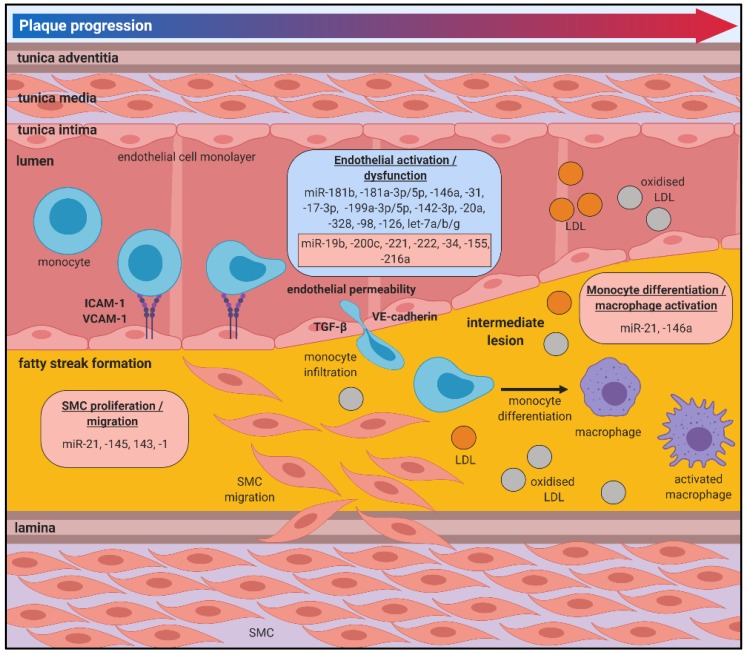
miRNAs in atherosclerotic plaque initiation and progression. Damage to the endothelial lining of lesion-prone areas of the arterial vasculature is one of the earliest events that contributes to the pathobiology of atherosclerosis, promoting a pro-inflammatory milieu and inducing an oxidative stress environment, which facilitates the recruitment of monocytes to the vessel wall via the increased expression of cell adhesion molecules (CAMs) including ICAM-1 and VCAM-1. Monocytes undergo trans-endothelial migration into the intima, where they undergo differentiation into macrophages and become activated under inflammatory and oxidative stress conditions. LDL diffuses into the intima and undergoes oxidative modification, and is likely to be taken up by the macrophages. Atherosclerotic lesion progression involves the proliferation and migration of smooth muscle cells (SMCs) into the intima. Positive/atheroprotective (blue) or negative/atherogenic (red) effects of miRNAs on the atherosclerotic processes are shown.

The initial stages of atherosclerosis are often asymptomatic and can develop over several decades without being detected or treated. There is growing evidence that miRNAs mediate key cellular and molecular processes related to this early stage, which could provide insight on early progression and facilitate targeted interventions to prevent plaque development (Figure 2).

### 4.1. Endothelial Dysfunction

Damage to the endothelial lining of lesion-prone areas of the arterial vasculature is one of the earliest events that contributes to the pathobiology of atherosclerosis, promoting a pro-inflammatory milieu and inducing an oxidative stress environment, which facilitates the recruitment of inflammatory cells to the vessel wall.

The nuclear factor (NF)ĸB signalling pathway is a critical driver of endothelial dysfunction and is activated by many risk factors that drive atherosclerosis including inflammatory cytokines including tumour necrosis factor (TNF)α, diabetes, oxidised (ox)LDL, angiotensin II and haemodynamic stress [45]. Upon activation, the NFĸB signalling drives the expression of endothelial leukocyte adhesion molecules including E-selectin, VCAM-1 and intracellular adhesion molecule (ICAM)-1. Numerous miRNAs have been implicated in the inflammatory response in endothelial cells. Members of the miR-181 family play an important role in suppressing endothelial inflammatory responses by targeting mediators of the NFĸB signalling pathway. miR-181b targets importin-α3, a protein that facilitates NFĸB nuclear translocation, inhibiting an enriched set of NFĸB-responsive genes including VCAM-1 and E-selectin [46]. Systemic miR-181b delivery reduces NFĸB activity and atherosclerotic lesion formation in *Apoe*^−/−^ mice [47]. miR-181a-5p and miR-181a-3p inhibit vascular inflammation through regulation of the NFĸB signalling pathway to inhibit VCAM-1, ICAM-1 and E-selectin expression [48]. Rescue of miR-181a-5p and miR-181a-3p significantly suppresses atherosclerotic plaque formation in *Apoe*^−/−^ mice [48]. miR-146a is involved in a negative feedback loop control of NFĸB signalling [49], by targeting TRAF6, which inhibits downstream IκB kinase (IKK) phosphorylation and nuclear translocation [50] to suppress ICAM-1 expression and macrophage migration [51]. miR-31 and miR-17-3p are involved in a negative feedback loop that directly inhibits TNFα-induced E-selectin and ICAM-1 expression [52]. miR-155 and miR-221/222 inhibit the angiotensin II-induced inflammatory response by targeting the transcription factor Ets-1 and its downstream genes, VCAM-1 and monocyte chemoattractant protein (MCP)-1 [53]. Finally, let-7g exerts anti-inflammatory effects by inhibiting transforming growth factor (TGF)-β signalling in endothelial cells while in vivo lentiviral delivery of let-7g inhibitors induced overgrowth of the carotid intima-media layer in mice [54].

The loss of nitric oxide (NO) bioavailability is central to endothelial dysfunction. Production of NO is facilitated by the endothelial nitric oxide synthase (eNOS) pathway. Oxidative stress is driven by an imbalance in favour of increased generation of reactive oxygen species (ROS) coupled with a reduction in the body’s innate antioxidant defence systems [55]. ROS production in the vessel wall is increased in all conditions considered risk factors for atherosclerosis including hypertension, diabetes, smoking and dyslipidaemia [56]. Increased ROS production drives endothelial injury by promoting increased inflammation, apoptosis, vascular permeability and LDL oxidation [57]. miRNAs that can mediate endothelial homeostasis through NO and ROS production within the vessel wall would be therapeutically beneficial. miR-199a-3p and miR-199a-5p were both shown to independently mediate NO bioavailability by promoting eNOS activity and reducing its degradation through the upregulation of phosphatidylinositol 3-kinase (PI3K)/protein kinase B (Akt) and calcineurin pathways and by targeting the antioxidant enzymes superoxide dismutase (SOD)1 and peroxiredoxin (PRDX)1 [58]. miR-200c is upregulated in response to oxidative stress and dysregulates the sirtuin 1 (SIRT1)/Forkhead box O1 (FOXO1)/endothelial nitric oxide synthase (eNOS) regulatory loop, controlling oxidative stress tolerance [59]. This in turn results in the decreased expression of the ROS scavengers, catalase and manganese superoxide dismutase (MnSOD), leading to increased ROS presence and decreased NO bioavailability. Recently, miR-142-3p has emerged as another miRNA that inhibits oxidative stress-induced endothelial cell apoptosis and atherosclerotic plaque development in mice by activating the Akt/eNOS pathway [60]. miR-19b, miR-221 and miR-222, which are highly expressed in the intima of atherosclerotic lesions, contribute to endothelial dysfunction by increasing ROS production through PPARG coactivator (PGC)-1α, a critical transcriptional regulator of energy metabolism and mitochondrial function [61]. Mitochondrial function plays a vital role in endothelial cell propensity towards damage and the insult of ROS on the endothelium. miR-20a and miR-328 reduced ROS production by targeting components of the toll-like receptor (TLR)4 inflammatory pathway [62,63].

Increased ROS production drives the oxidation of LDL, a critical early event in atherosclerosis that contributes to lipid accumulation. The main oxLDL receptor, lectin-like oxidised low-density lipoprotein receptor-1 (LOX-1), is upregulated in response to pro-inflammatory and pro-atherogenic stimuli and is known to drive endothelial activation and in foam cell formation. Both miR-98 and let-7a/b inhibit LOX-1 expression, which prevents oxLDL uptake into the endothelium [64,65]. Conversely, miR-34 antagonises oxLDL cell injury by targeting the cell-survival gene bcl-2 [66]. 

Modification to endothelial barrier integrity further contributes to trans-endothelial migration of monocytes through the intima. In addition to its anti-inflammatory effects, miR-155 promotes endothelial permeability by inhibiting zonula occludens-1 (ZO-1), claudin 1, β-catenin and VE-cadherin, which modifies endothelial barrier function by destroying tight junctions [67]. This was shown to be mediated via VSMC-derived miR-155, which was secreted in exosomes by VSMCs following oxLDL treatment, exhibiting a communicative interaction between endothelial cells and VSMCs in response to atherogenic stimuli. Conversely, miR-126 inhibits endothelial permeability by targeting TGF-β [68], which dampens the inflammatory response and decreases endothelial damage via ROS.

Ageing is an important risk factor for the development of atherosclerosis and is accompanied by the decline of endothelial function. Senescence of endothelial cells has been proposed to be involved in endothelial dysfunction and atherogenesis. Exploration of miRNAs implicated in endothelial senescence could facilitate new approaches to reverse or dampen the effects of ageing. In addition to its anti-inflammatory effects, let-7g also targets endothelial senescence by regulating the SIRT1 and the insulin growth factor (IGF)1 pathway [69]. miR-216a was shown to promote premature endothelial senescence via the transforming growth factor (TGF)-β1 pathway [70].

The compounding effects of endothelial activation and the recruitment of pro-inflammatory mediators within the intima is devastating to vascular function. These events promote a sustained pro-inflammatory phenotype within the vascular endothelium that becomes progressively detrimental, contributing to endothelial senescence and plaque progression. Controlled regulation of endothelial activation by miRNAs hold therapeutic potential in the early intervention and targeted control of atherosclerosis.

### 4.2. Monocyte Recruitment, Macrophage Differentiation and Foam Cell Formation

Immune responses to the pro-inflammatory environment within the vessel wall also contribute critically to atherosclerosis. Monocytes are recruited to “clean up” lipids in response to chemoattractant signals and once in the intima they differentiate into macrophages [71]. Macrophages are important in the pathophysiology of atherosclerosis by maintaining vessel wall lipid homeostasis and secreting inflammatory mediators. Within the plaque area, macrophages take up lipids resulting in the formation of lipid-laden inflammatory foam cells. miRNAs have been shown to regulate key macrophage processes that underpin atherosclerotic progression (Figure 2).

**Figure 3:**
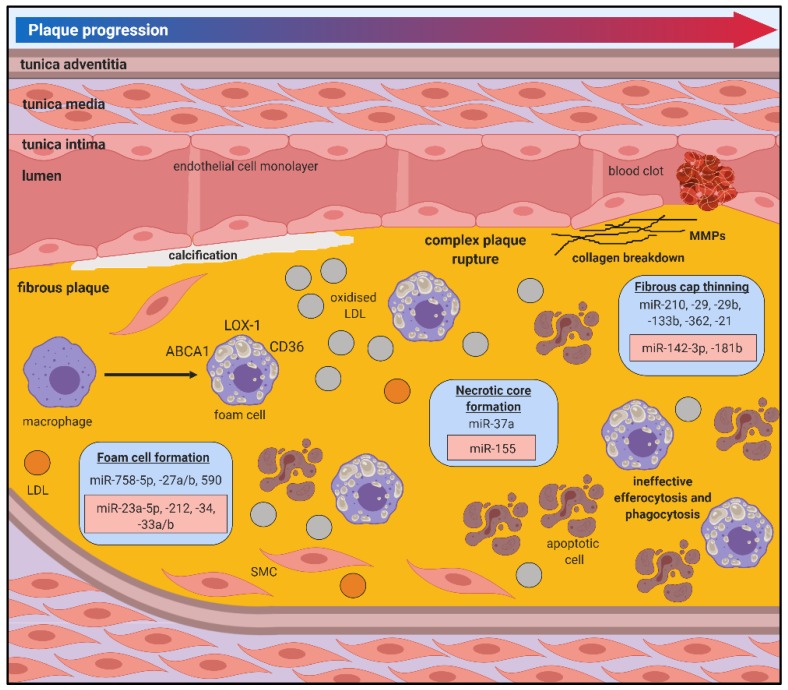
miRNA regulation of atherosclerotic plaque rupture. Rupture-prone vulnerable atherosclerotic plaques typically consist of high inflammatory cell content (foam cells) and a large necrotic core covered by a thin fibrous cap. These vulnerable plaques have an increased susceptibility to rupture, often culminating in catastrophic clinical manifestations of myocardial infarction or ischaemic stroke. While the pathophysiology of plaque rupture is not fully understood, it is well accepted that lesion vulnerability is more closely associated with plaque composition than size. Positive/atheroprotective (blue) or negative/atherogenic (red) effects of miRNAs on the atherosclerotic processes are shown.

miR-21 drives leukocyte polarisation and survival under inflammatory conditions [72]. In the early stages of atherosclerosis, the absence of miR-21 decreased the availability of circulating monocytes and increased apoptosis of plaque macrophages, which limited lesion development [73]. miRNAs are differentially expressed during foam cell formation in vitro (Figure 3) [74]. Plaque macrophages express CD36, which facilitates the uptake of LDL with only minor oxidative modifications, making it particularly potent in the context of atherosclerosis [75]. miR-758-5p reduces macrophage oxLDL uptake by directly inhibiting expression of the scavenger receptor CD36 [76]. miR-27a/b regulates macrophage cholesterol homeostasis by targeting genes involved in cholesterol uptake (LOX-1 and CD36) and efflux (ABCA1) [77]. miR-23a-5p and miR-212 increase foam cell formation by reducing cholesterol efflux from macrophages through ABCA1 [31,78]. miR-34 increases the binding capacity of oxLDL to macrophages by facilitating bridging between lipoprotein lipase (LPL) and LOX-1 [79]. Conversely, miR-27 and miR-590 inhibit LPL expression to prevent macrophage lipid accumulation and cytokine release [77,80,81].

Macrophages can also secrete extracellular vesicles containing miRNAs that in turn exert downstream cellular effects. miR-146a was the most abundant miRNA found in vesicles secreted by macrophages exposed to oxLDL [82]. Furthermore, miR-146a enriched vesicles diminished macrophage migration by inhibiting insulin like growth Factor 2 mRNA binding protein 1 (IGF2BP1) and human antigen R (HuR), suggesting that these effects could prevent macrophage emigration from atherosclerotic lesions [82].

Differentiation of monocytes to macrophages is driven by two key colony stimulating factors (CSF), macrophage (M)-CSF and granulocyte macrophage (GM)-CSF, which induce both divergent and convergent alterations in gene expression [83]. To date, there is no known miRNA that directly targets the GM-CSF receptor [84]. Several miRNAs that are direct targets of M-CSF, including mir-22, miR-34a and miR-155 [85]. Whilst one study described that 87% of gene expression alterations were conserved between M-CSF and GM-CSF, the role of miRNAs involved in monocyte-to-macrophage polarisation remains to be fully explored. In atherosclerotic lesions, macrophages are exposed to a wide range of microenvironmental signals, which influence macrophage polarisation towards either the classical M1 pro-inflammatory phenotype or alternatively activated M2 anti-inflammatory reparative macrophages [86]. Studies have identified the differential expression of miRNAs during M1/M2 polarisation: miR-155-5p, miR-181a and miR-451 are associated with M1 macrophages, whilst expression of miR-125a, miR-145-5p and miR-146a are elevated in M2 macrophages. Furthermore, knockout of miR-155 impaired M1 polarisation, while miR-155 overexpression reprogrammed M2 to M1 macrophages [87] and promoted the accumulation of M1 macrophages [88]. miR-33 is known to regulate macrophage phenotype by sustaining inflammatory M1 macrophages [89]. With inhibition of miR-33 inducing metabolic reprogramming of atherosclerotic plaque macrophages towards the M2 phenotype, driving tissue repair and reducing inflammation. Whilst there remains an attractive avenue for the therapeutic potential of miRNA mimics/inhibitors to modulate macrophage polarisation, more research is needed to understand the complex interplay between miRNAs and inflammation. 

### 4.3. Vascular Smooth Muscle Cell Proliferation and Differentiation

Vascular smooth muscle cells (VSMCs) are the most abundant cell type in the arterial wall and are involved throughout atherosclerosis progression. EC loss due to apoptosis, stenting or mechanical forces can promote VSMC migration from the media to the intima of the vessel well, where they then proliferate to promote formation of the neointima and plaque, contributing to atherosclerosis progression [90]. Atherogenic stimuli contribute to a switch in VSMC phenotype from contractile to synthetic, which is characterised by changes in the proliferative, migrative and apoptotic nature of the cells.

miRNAs have been shown to target numerous transcription factors, signalling cascades and growth factors that regulate VSMC proliferation (Figure 2) [91]. A recent study conducted in SMCs isolated from human saphenous veins showed that miR-21 overexpression increases the proliferative capacity of these cells and drives the switch towards a more synthetic phenotype [92]. Taken in tandem with its effects on leukocytes, this highlights the importance of miR-21 in the early stages of atherosclerosis. The miR-143/145 cluster are established modulators of VSMC plasticity, driving the more proliferative SMC phenotype by cooperatively mediating a transcriptional network including Kruppel like factor (KLF)5, KLF4 and myocardin [93,94]. These phenotypic changes lead to the development of macrophage-like characteristics and oxLDL uptake by VSMCs [95]. miR-145 was further implicated in promoting changes to VSMC contractile markers including α-smooth muscle actin and calponin [96]. Additionally, miR-1 is shown to mediate changes to contractile proteins in VSMCs including suppression of α-smooth muscle actin [97].

In general, VSMC are thought to contribute to plaque stability via mediating extracellular matrix (ECM) structural elements. However, once undergoing phenotypic changes, VSMCs also contribute to necrotic core development and fibrous cap thinning. miRNAs that mediate changes to the VSMC phenotype by targeting molecules involved in vessel contraction and SMC proliferation and migration could be potential targets to reverse or inhibit the contributions of VSMCs in atherosclerotic plaque progression.

## 5. MiRNAs in Atherosclerotic Plaque Rupture

Rupture-prone, vulnerable atherosclerotic plaques typically consist of high inflammatory cell content and a large necrotic core covered by a thin fibrous cap. These vulnerable plaques have an increased susceptibility to rupture, often culminating in catastrophic clinical manifestations of myocardial infarction or ischaemic stroke. While the pathophysiology of plaque rupture is not fully understood, it is well accepted that lesion vulnerability is more closely associated with plaque composition than size. There is increasing evidence that miRNAs regulate processes associated with plaque rupture (Figure 3).

### 5.1. Fibrous Cap Thinning

Local factors regulating SMC proliferation and apoptosis in the vessel wall contribute to the stability of the atherosclerotic fibrous cap, with vulnerable plaques showing increased evidence of SMC death and decreased SMC numbers in the fibrous cap. Numerous miRNAs shown to target SMCs in atherosclerotic plaques could have therapeutic potential in stabilising vulnerable plaques. miR-21 overexpression inhibits ROS-induced SMC apoptosis in vitro [98] while local delivery of a miR-21 mimic into carotid plaques rescued the vulnerable plaque rupture phenotype by increasing SMC proliferation [99]. Systemic miR-210 overexpression increased the stability of the fibrous cap of carotid atherosclerotic lesions in *Apoe*^−/−^ mice by promoting SMC proliferation and survival [100].

Breakdown of ECM components and the dysregulation of processes involved in regulating collagen structure and content, ECM synthesis and inflammation contribute to fibrous cap thinning and plaque vulnerability [101]. Enhancement of plaque ECM may improve plaque morphology and stabilise lesions, reducing the risk of plaque rupture. Overexpression of miR-124-3p reduced atherosclerotic plaque stability in *Apoe*^−/−^ mice by decreasing VSMC content and inhibiting type I and type III collagen expression [102]. Conversely, inhibition of miR-29 promoted the expression of ECM genes (Col1A and Col3A) and collagen and elastin content, which resulted in reduced lesion size, increased fibrous cap thickness and reduced necrotic zones [103].

ECM structure is also mediated by a network of regulatory elements including matrix metalloproteinases (MMPs) [101]. MMPs contribute to plaque stability by catalysing reactions that either disintegrate or stabilise ECM components. In addition to its effects in regulating ECM content, miR-29b also inhibits MMP2 expression [104]. The atherogenic properties of VSMCs are decreased by miR-133b inhibition, which targets MMP9 [105], a key regulator of collagen degradation that is significantly upregulated in atherosclerotic plaques [106]. Tissue inhibitors of MMPs (TIMPs) regulate the action of MMPs and contribute to maintaining ECM content and plaque stabilisation [101]. Inhibition of miR-181b in *Apoe*^−/−^ and *Ldlr*^−/−^ mice promoted the stabilisation of existing plaques by targeting TIMP3 and VSMC elastin production [107]. miR-362 inhibits A disintegrin and metalloproteinases with thrombospondin domains (ADAMTS)1, another metalloproteinase that destabilises the ECM by cleaving proteoglycans and has been implicated in atherosclerosis [108]. 

### 5.2. Necrotic Core Formation

Necrotic core formation is facilitated by cellular apoptosis and death of macrophages/foam cells due to ineffective efferocytosis and an imbalance in healthy versus apoptotic macrophages [109]. Cells undergoing apoptosis secrete signals that facilitate uptake by phagocytic cells. These signals can be mediated by miRNAs to cause shifts in the proportion of healthy versus apoptotic macrophages present within the plaque. miR-378a regulates signal regulatory protein (SIRP)α-mediated phagocytosis and polarisation of macrophages, which contribute to imbalances in efferocytosis in the plaque [110]. In addition to its effects in other cell types, miR-155 was shown to have divergent stage-specific effects in lesion formation. miR-155 knockout in *Apoe*^−/−^ mice enhanced lesion formation, increased lesional macrophage content and promoted macrophage proliferation after 12 weeks of the high-cholesterol diet. However, inhibition of miR-155 reduced necrotic core formation and the deposition of apoptotic cell debris, preventing the progression of atherosclerosis between 12 and 24 weeks of the high-cholesterol diet [111]. These studies highlight the complex and layered regulatory networks of disease progression that is controlled by miRNAs, highlighting the need to comprehensively explore the role of key miRNAs and their effects at various stages of disease progression to provide an insight on the best approach to capitalise on their therapeutic capacity.

## 6. MiRNAs in Atherosclerotic Plaque Neovascularisation

### 6.1. Vasa Vasorum Formation and Vascular Remodelling

**Figure 4:**
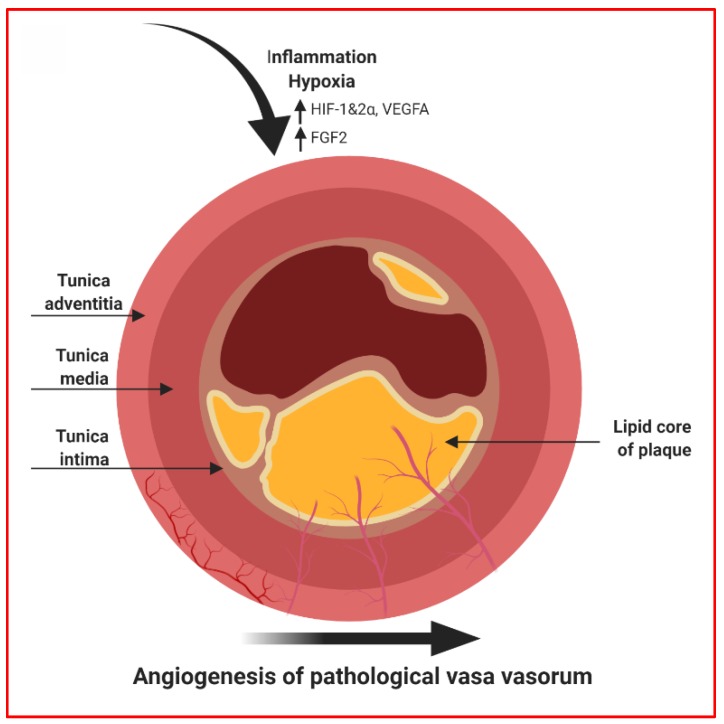
Disruption of physiological vasa vasorum contributes to plaque formation. Factors such as diabetes, hypertension and hypercholesteraemia can lead to localised or systemic inflammation and hypoxia driving atherogenic conditions. Formation of adventitial vasa vasorum (VV) occurs in response to the metabolic demand of the outer and medial layers of an artery. Under hypoxic conditions, hypoxia-inducible factor (HIF)-1α and HIF-2α induce vascular endothelial growth factor (VEGF)A, a proangiogenic mediator. Hypoxic conditions also provide favourable conditions for fibroblast growth factor (FGF)2, promoting EC growth and stabilising VV. Additionally, inflammation triggers VV sprouting from the adventitia into the arterial lumen by inducing secretion of several angiogenic growth factors.

Although often viewed simplistically as the build-up of cholesterol-rich plaques in the subintimal compartment of the vessel wall, the pathology of atherosclerosis is in fact transmural. In addition to the “inside-out” recruitment of circulating lipoproteins and inflammatory cells from the vessel lumen into plaque, converging lines of evidence point to a parallel “outside-in” pathway to atherogenesis. This involves early and rapid remodelling of the outer vascular layer, or adventitia, consisting of expansion of inflammatory cells and permeable microvessels, called *vasa vasorum* (VV), that infiltrate and progressively destabilise the growing plaque [112]. VV are a specialised microvasculature that supply the adventitia and outer media layer of the vessel with oxygen and nutrients under normal physiologic conditions (Figure 4) [113]. 

Accumulating evidence shows that changes in VV characteristics are closely associated with the progression of atherosclerosis [114]. The focal expansion of VV precedes and co-localises with atherosclerotic lesions, with their density correlating strongly with plaque area [115,116,117,118]. VV infiltrate plaque as immature, leaky microvessels that exacerbate the deposition of pro-inflammatory cells and particles, while also contributing to plaque haemorrhage [119]. As plaque grows, a hypoxic gradient is created across the thickened artery wall which further stimulates neoangiogenesis of VV. Given their key pathogenic roles, the inhibition and stabilisation of VV have emerged as enticing therapeutic objectives to favourably modify plaque and address the unacceptable burden of atherosclerotic cardiovascular disease that persists despite current treatments [120]. Accumulating evidence has also revealed important regulatory roles of miRNAs in the aberrant formation of VV in atherosclerotic arteries [121]. It has been postulated that multiple miRNAs may govern how adventitial progenitor cells normally regulate VV development [122,123], while altered miRNA expression may result in abnormal VV formation and expansion in atherosclerotic arteries [112].

### 6.2. Vascular-Resident Stem Cell Differentiation and Neovascularisation

**Figure 5:**
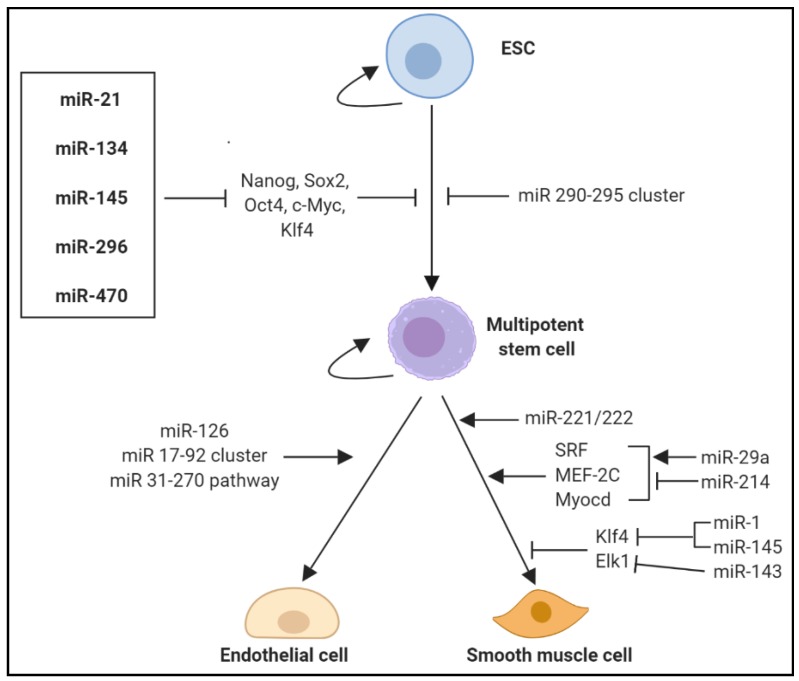
Role of miRNAs in regulation and self-renewal of embryonic stem cells and differentiation of stem/progenitor cells into endothelial and smooth muscle cell lineages. Multiple miRNAs are involved in regulating ESC differentiation into multipotent stem/progenitor cells, largely by targeting stemness factors. miR-21, miR-134, miR-145, miR-296 and miR-470 promote ESC differentiation by inhibiting transcription factors Nanog, Sox2, Oct4, c-Myc and Klf4. In contrast, the miR 290-295 cluster inhibits progression, termed embryonic stem cell-specific cell cycle-regulating miRNAs. Other key miRNAs promote cardiovascular lineage differentiation and regulate cell phenotype, including endothelial and smooth muscle cell commitment.

Additionally, the VV serve as the vascular niche for vascular-resident stem cells (VSCs), acting as a stem cell reservoir to supply VSCs, which can differentiate into ECs and VSMCs, into the intima, contributing to atherosclerotic re-modelling [114]. VSCs have potent angiogenic effects through their paracrine properties and/or ability to differentiate into ECs or SMCs, thereby contributing to the growth of the VV within atherosclerotic lesions. Controlled regulation of stem cell differentiation into cardiovascular lineages cells would dampen the influence of VV neovascularisation in the progression of atherosclerosis. Several miRNAs have been found to mediate embryonic stem cell (ESC) differentiation and self-renewal into specific cell lineages, including different vascular, endothelial and haematopoietic cell types (Figure 5). Numerous miRNAs including miR-21, miR-134, miR-145, miR-296 and miR-470 promote ESC differentiation by targeting transcription factors that drive stemness including Nanog, Sox2, Oct4, c-Myc and Klf4 while the miR-290-295 cluster has been shown to inhibit ESC differentiation [124]. Furthermore, several miRNAs play an important role in the differentiation of cardiovascular lineage cells including ECs and SMCs.

Multiple miRNAs have been shown to regulate endothelial cell (EC) commitment and vasculogenic growth, including that of the VV. The importance of miRNAs in vascular development and angiogenesis was first observed when the enzyme Dicer was inhibited with embryonic lethality observed during early development due to an underdeveloped vascular system [125,126]. miR-126 is involved in regulating angiogenic signalling and vessel integrity and is significantly upregulated in vasculogenic progenitors when compared to undifferentiated ESCs [127]. miR-126 has atheroprotective properties under normal homeovascular conditions, suppressing the inflammatory cascade and mediating leukocyte adherence in atherosclerosis by decreasing VCAM-1 expression [128] and inhibiting Sprouty related EVH1 domain containing 1 (SPRED1) and phosphoinositide-3-kinase regulatory subunit 2 (PIK3R2), inhibitors of vascular endothelial growth factor (VEGF)A signalling [129]. miR-126^−/−^ mice have a reduced ability to survive myocardial infarction due to vascular abnormalities and defective angiogenesis [130]. miR-126 is aberrantly expressed under hypoxic conditions, causing an over-abundant formation of neovessels [131]. Given the hypoxic nature of the atherosclerotic milieu into which the VV expand, miR-126 upregulation may result in excessive angiogenesis and localised proliferation of unstable neovessels, promoting the pro-atherogenic response, highlighting the importance of miR-126 in regulating adaptive and maladaptive angiogenic responses. The miR-17–92 cluster is highly expressed during developmental vasculogenesis in both embryonic and post-natal cell lineages [132]. Extensive studies have established the anti-angiogenic role of miR-92a [133,134], with miR-92a inhibition shown to enhance angiogenesis and accelerate re-endothelialisation [135]. The miR-31–miR-720 pathway is critical in EPC activation and downregulation of this pathway contributes to the pathogenesis of CAD. Overexpression of miR-31 rescued angiogenic and vasculogenic abilities of EPCs derived from patients with CAD by targeting thromboxane A2 receptor (TBXA2R) and FAT atypical cadherin 4 (FAT4), which in turn regulates VEGFA directly or indirectly via hypoxia-inducible factor (HIF)-1α [136]. FAT4 in turn induces the expression of miR-720, which inhibits VASH1, a VEGFA-inducible secreted protein that inhibits angiogenesis and vascular function by inducing prolyl hydroxylase–mediated degradation of HIF-1α [136]. Endothelial progenitor cells (EPCs) mediate and promote EC growth. In addition to its effects on leukocytes and smooth muscle cells, miR-21 levels are strikingly upregulated in EPCs from CAD patients [137]. Furthermore, miR-21 overexpression was shown to inhibit EPC migration while miR-21 inhibition restored EPC function [137].

Numerous miRNAs have also been implicated in SMC differentiation. In addition to their role as modulators of VSMC plasticity, miR-143 and miR-145 are important mediators in the differentiation of multipotent stem cells into the VSMC phenotype [93]. Dicer knockout of miR-143 and miR-145 proved lethal to mouse embryos due to extensive vessel haemorrhage and thin-walled aortas that was primarily attributed to decreased VSMC proliferation [138]. Furthermore, fewer progenitor cells committed towards the VSMC phenotype and a subsequent loss of smooth muscle contractile function in many tissues. miR-145 and miR-143 cooperatively target a network of transcription factors, including Klf4 and Elk-1 to promote SMC differentiation and directing smooth muscle fate [93]. miR-1 has also been shown to regulate ESC/progenitor cell differentiation into VSMCs. miR-1 expression is upregulated and highly enriched during ESC differentiation to VSMCs, while repression of miR-1 inhibited VSMC phenotype commitment and differentiation [139,140]. miR-1 was shown to inhibit Klf4, a key transcription factor in the regulation of VSMC proliferation, differentiation, apoptosis and cell reprogramming [140]. miR-221 and miR-222 are two highly homologous miRNAs enriched in both ECs and VSMCs [141,142] and are postulated to contribute to VV expansion by acting on progenitor cell populations seeded in vessel walls. miR-221 and miR-222 have distinct and opposing effects on VSMCs and ECs, promoting proliferation in VSMCs but initiating EC death pathways and quiescence [141]. miR-221 and miR-222 have been shown to influence the angiogenic capacity of ECs [143] and phenotypic properties of VSMCs [144]. miR-29a has also been shown to mediate VSMC differentiation from ESCs by targeting SRF, MEF-2C and myocardin (Myocd), three well-known transcription factors for VSMC gene regulation [145]. miR-214 regulates VSMC differentiation from ESCs by suppressing Quaking (QKI), which downregulates the expression of SRF, MEF-2C and Myocd [146].

Collectively, these studies highlight the role of miRNAs in both the regulation of cardiovascular lineage differentiation and angiogenesis. Under pathophysiological conditions, this may drive the formation of new vessels, including VV into neointimal lesions. While these studies suggest the potential targeting of miRNAs in this space, additional research is required to unravel the involvement of miRNAs in VV growth during atherogenesis.

## 7. Therapeutic Potential of Targeting MiRNAs to Treat Atherosclerosis

Table 1 provides an overview of the different cell types and cellular processes involved in the progression of atherosclerosis targeted by the miRNAs summarised in this review. Several miRNAs have been shown to simultaneously regulate different processes across multiple cell types. This highlights the therapeutic potential for targeting miRNAs in order to provide a holistic approach for the prevention of atherosclerosis. For example, miR-21 drives critical stages at the initiation phase of atherosclerosis including leukocyte polarisation and survival, smooth muscle cell proliferation and differentiation towards a synthetic phenotype, embryonic stem cell differentiation and endothelial progenitor cell migration and function. Additionally, miR-34 has been shown to confer protection to the endothelium against oxidative stress and is also important in macrophage differentiation and foam cell formation. miR-155 also has the capacity to exert simultaneous effects on multiple properties that influence endothelial dysfunction, macrophage differentiation with divergent stage-specific effects in macrophages within the atherosclerotic lesion. The miR-143/miR-145 cluster are critical modulators of VSMC maintenance and proliferation and can not only drive the differentiation of multipotent stem cells towards the VSMC phenotype but are also important in modulating VSMC plasticity, which could have significant influence on the vessel wall. Furthermore, miRNAs can have distinct and opposing effects on different cell types, as evidenced by the two highly homologous miRNAs, miR-221 and miR-222, and their ability to promote VSMC proliferation while concurrently driving EC apoptosis. Further exploration on the expansive role of these miRNAs as well as other emerging miRNAs will provide insight on the most effective way to fully capitalise on their properties in a therapeutic manner.

## 8. Circulating MiRNAs as Potential Clinical Biomarkers for Atherosclerosis and CVD

Extensive research has revealed that miRNAs are extremely stable and expressed in the peripheral circulation. miRNAs can be easily obtained and are reliably detected from plasma or serum by non-invasive methods [147]. Given that miRNAs are differentially regulated at various stages in the pathophysiology of atherosclerosis in a tissue- and cell-specific manner, this has opened a new paradigm for their use as diagnostic biomarkers to improve differential clinical diagnosis from subclinical atherosclerotic disease to acute coronary syndromes. Several studies have explored miRNA profiles in correlation with atherosclerotic disease burden.

In a human study of advanced coronary atherosclerotic plaques, miR-21, miR-92a and miR-99a were found to be upregulated in the circulation [148]. Consistent with this, miR-21 is significantly upregulated in serum levels of patients presenting with clinical atherosclerosis [149]. However, only miR-21 levels were upregulated in symptomatic compared to asymptomatic plaques, suggesting that miR-21 may be a better biomarker that correspondingly reflects symptomatic plaque burden [148]. A recent systematic review of 18 studies identified a common miRNA profile (deregulation of miR-21, miR-30, miR-126 and miR-221-3p) that is associated with different atherosclerotic disease locations [150]. Specific miRNA patterns were also identified for each territory: miR-21 and miR-29 were found in carotid atherosclerosis while let-7e, miR-27b, miR-130a and miR-210 were deregulated in lower limb atherosclerosis [150]. Assessment of downstream cellular functions found that these deregulated miRNAs are associated with control of angiogenesis, endothelial cell function, inflammation, cholesterol metabolism, oxidative stress and ECM composition, all of which underpin the pathophysiology of atherosclerosis. Studies have also reported elevated levels of circulating miR-221 and miR-222 in patients with clinical atherosclerosis, which were positively correlated with triglyceride and VLDL levels [8,149].

Circulating miRNAs were also altered in response to established CAD. Patients with CAD had reduced levels of VSMC-enhanced miR-145; EC-enriched miRNAs miR-126, miR-17 and miR-92a; and miR-155, yet expression of cardiomyocyte-enriched miRNAs miR-133 and miR-208a was elevated [151]. CAD is associated with a significant decrease of circulating miRNAs that are preferentially expressed in ECs and VSMCs, whereas prototypic muscle-enriched miRNAs are increased, which may be caused by an uptake of circulating miRNAs into atherosclerotic plaques or lesions, further contributing to the neovascularisation and inflammatory progression of the plaque. Other studies have also reported that circulating miR-19a, miR-29a, miR-30e, miR-145, miR-150, miR-155, miR-181d, miR-342, miR-378 and miR-484 levels are decreased in patients with stable CAD [152] and has been associated with subclinical atherosclerosis [152]. CAD severity was found to be inversely associated with circulating miR-126 levels [153]. Studies in patients with acute myocardial infarction (AMI) have identified several miRNAs which have the potential to act as predictive biomarkers. Circulating levels of miR-133 and miR-328 were strikingly increased (11–16 times) in patients with AMI [154]. miR-133 was increased by 12-fold approximately 2 h after AMI onset, typically earlier than most traditional AMI markers [155,156]. Circulating miR-21-5p and miR-361-5p rapidly increased after the onset of AMI symptoms, followed by a gradual decline in the following days, mimicking the dynamic trends seen with plasma cardiac troponin in early phase AMI [157]. miR-499, which is produced almost exclusively in the heart before entering the circulation [158], is proving to be a promising AMI diagnostic biomarker [158,159,160]. Multiple studies investigating patients diagnosed with AMI showed a significant elevation of plasma miR-499 as early as 1 h after onset of chest pain associated with AMI, with individuals in other CVD groups displaying non-detectable levels of miR-499 [159]. miR-223 is also known to reliably predict AMI in individuals with ACS, with five-fold elevations in serum miR-223 levels observed in AMI patients when compared to controls [161].

Studies have also reported that some miRNAs have differential expression patterns depending on the disease pathology. miR-126 levels are elevated in patients with atrial fibrillation and heart failure [162], while conversely low levels are reported in patients with angina or AMI [151,163,164], highlighting a diverse role for miR-126 in the cardiovascular system. Circulating levels of several 14q32 miRNAs (miR-134, miR-328, miR-370, miR-480 and miR-487a/b) also possess diagnostic value for CAD, AMI and cardiac death, respectively [161,165,166,167]. In patients with acute ischaemic stroke, increased levels of miR-487b were found in circulating leukocytes [168]. Other 14q32 miRNAs, including miR-541 and miR-665, have been described in cardiac hypertrophy and heart failure [169,170]. The 14q32 miRNA cluster has established roles in vascular remodelling by possessing both anti-angiogenic and pro-atherogenic properties, which could underpin the variations in differential expression patterns. Overall, the profiling of specific circulating miRNAs in atherosclerotic and wider CVD possesses promising research potential. Additional studies investigating their roles as definitive diagnostic and prognostic biomarkers in large prospective cohorts are now needed.

## 9. Conclusions

There has been significant therapeutic advancement in the treatment of atherosclerosis. However, it is evident that there remains a gap where novel therapeutic approaches can complement current therapies such as lipid-lowering drugs (e.g., statins and fibrates) and anti-hypertensive drugs to provide a holistic approach for treating atherosclerosis to orchestrate the regulation of complex signalling networks across multiple cell types and different stages of disease progression. Since their discovery in 2000, there has been significant interest in the potential of miRNAs as both therapeutic targets and clinical diagnostic biomarkers in multi-faceted diseases including atherosclerosis. In vitro and pre-clinical studies to date show that numerous miRNAs have the potential to regulate complex cellular processes across key cardiovascular cell types. We strongly believe that this highlights their clinical potential as emerging therapeutics that can be customised as personalised treatments to specifically target the different stages of atherosclerosis progression. Furthermore, targeted modulation of miRNAs has the capacity to be highly effective for the treatment of atherosclerosis because of their ability to orchestrate a host of complex factors concurrently across multiple cell types. The field of miRNA biology has expanded considerably over the last 20 years and will continue to grow rapidly as miRNA therapeutics enter clinical trials. Furthermore, growing evidence suggest that circulating miRNAs can provide insight into a patients’ specific atherosclerotic disease stage to better inform clinical diagnosis. This highlights the significant potential for the use of miRNAs as theranostic targets, providing an attractive therapeutic approach to facilitate interventions targeted at patient-specific stages of atherosclerosis and the future management of its cardiovascular complications.

## Figures and Tables

**Table 1:** Summary of the miRNAs highlighted in this review and their effects on different cell types and cellular processes involved in the progression of atherosclerosis.

Stage of Disease	Cellular Process	miRNA	Cell Type
Cholesterol homeostasis and reverse cholesterol transport	Cholesterol biosynthesis	miR-24 miR-122 miR-185 miR-223 miR-486	Hepatocytes
	Cholesterol efflux	miR-10b miR-20a/b miR-23a/b miR-30e miR-33a/b miR-92a miR-101 miR-144 miR-148 miR-302a	Macrophages
Atherosclerotic plaque initiation and progression	Inflammation	let-7g miR-17-3p miR-31 miR-146a miR-155 miR-181a-3p/-5p miR-181b miR-221 miR-222	Endothelial cells
	Oxidative stress	let-7a/b miR-19b miR-20a miR-34 miR-98 miR-142-3p miR-199a-3p/-5p miR-200c miR-221 miR-222 miR-328	Endothelial cells
	Modification of endothelial barrier integrity	miR-155	Endothelial cells
	Senescence	let-7g miR-216a	Endothelial cells
	Monocyte recruitment	miR-21	Monocytes
	Monocyte to macrophage differentiation	miR-22 miR-27a/b miR-33 miR-34a miR-155	Monocytes
	Macrophage polarisation	miR-33 miR-155	Macrophages
	Foam cell formation	miR-23a-5p miR-27 miR-34 miR-212 miR-590 miR-758-5p	Macrophages
	Vascular smooth muscle cell proliferation and differentiation	miR-1 miR-21 miR-143/145	Smooth muscle cells
Atherosclerotic plaque rupture	Fibrous cap thinning	miR-21 miR-29b miR-124-3p miR-133b miR-181b miR-362	Smooth muscle cells
	Necrotic core formation	miR-155 miR-378a	Macrophages
Atherosclerotic plaque neovascularisation	Embryonic stem cell differentiation	miR-21 miR-134 miR-145 miR-290/295 miR-296 miR-470	Embryonic stem cells
	Endothelial cell commitment and vasculogenic growth	miR-17-92 cluster miR-21 miR-31 miR-126 miR-720	Multipotent stem cells
	Smooth muscle cell differentiation	miR-1 miR-29a miR-143 miR-145 miR-214 miR-221/222	Multipotent stem cells
